# Identification of mutated core cancer modules by integrating somatic mutation, copy number variation, and gene expression data

**DOI:** 10.1186/1752-0509-7-S2-S4

**Published:** 2013-10-14

**Authors:** Junhua Zhang, Shihua Zhang, Yong Wang, Xiang-Sun Zhang

**Affiliations:** 1 National Center for Mathematics and Interdisciplinary Sciences, Academy of Mathematics and Systems Science, Chinese Academy of Sciences, Beijing 100190, China

## Abstract

**Motivation:**

Understanding the molecular mechanisms underlying cancer is an important step for the effective diagnosis and treatment of cancer patients. With the huge volume of data from the large-scale cancer genomics projects, an open challenge is to distinguish driver mutations, pathways, and gene sets (or core modules) that contribute to cancer formation and progression from random passengers which accumulate in somatic cells but do not contribute to tumorigenesis. Due to mutational heterogeneity, current analyses are often restricted to known pathways and functional modules for enrichment of somatic mutations. Therefore, discovery of new pathways and functional modules is a pressing need.

**Results:**

In this study, we propose a novel method to **i**dentify **M**utated **C**ore **M**odules in **C**ancer (iMCMC) without any prior information other than cancer genomic data from patients with tumors. This is a network-based approach in which three kinds of data are integrated: somatic mutations, copy number variations (CNVs), and gene expressions. Firstly, the first two datasets are merged to obtain a mutation matrix, based on which a weighted mutation network is constructed where the vertex weight corresponds to gene coverage and the edge weight corresponds to the mutual exclusivity between gene pairs. Similarly, a weighted expression network is generated from the expression matrix where the vertex and edge weights correspond to the influence of a gene mutation on other genes and the Pearson correlation of gene mutation-correlated expressions, respectively. Then an integrative network is obtained by further combining these two networks, and the most coherent subnetworks are identified by using an optimization model. Finally, we obtained the core modules for tumors by filtering with significance and exclusivity tests. We applied iMCMC to the Cancer Genome Atlas (TCGA) glioblastoma multiforme (GBM) and ovarian carcinoma data, and identified several mutated core modules, some of which are involved in known pathways. Most of the implicated genes are oncogenes or tumor suppressors previously reported to be related to carcinogenesis. As a comparison, we also performed iMCMC on two of the three kinds of data, i.e., the datasets combining somatic mutations with CNVs and secondly the datasets combining somatic mutations with gene expressions. The results indicate that gene expressions or CNVs indeed provide extra useful information to the original data for the identification of core modules in cancer.

**Conclusions:**

This study demonstrates the utility of our iMCMC by integrating multiple data sources to identify mutated core modules in cancer. In addition to presenting a generally applicable methodology, our findings provide several candidate pathways or core modules recurrently perturbed in GBM or ovarian carcinoma for further studies.

## Background

Cancer is a complex disease and multiple factors including genomic, epigenomic, and gene expression aberrations are involved in its formation and development [1]. Understanding the pathogenesis of cancer at the molecular level is a great challenge and will shed lights on the effective diagnosis and treatment of cancer patients. Rapid advances in high-throughput sequencing technologies create opportunities to address this task. Large-scale cancer genomics projects, such as the Cancer Genome Atlas (TCGA) [2], International Cancer Genome Consortium (ICGC) [3] and the Catalogue Of Somatic Mutations In Cancer (COSMIC) [4], have produced a large volume of data in recent years, providing a basis for systems level understanding of cancer formation and progression [5].

In general, cancer genomes possess a large number of mutations including somatic mutations and copy number variations (CNVs). Among them, some mutations contributing to cancer progression from the normal to the malignant state are called driver mutations, and those that accumulate in cells but do not contribute to cancer development are called passengers [6,7]. Therefore, distinguishing the functional driver mutations, driver pathways or core modules from random passengers will be a crucial step in understanding the molecular mechanisms of carcinogenesis, which can further aid in effective diagnosis, treatment and prognosis of cancer patients.

Initially, efforts were devoted to detect individual driver genes that cause tumors. A standard approach for this is to identify recurrent mutations in a large cohort of cancer patients. But the extensive mutational heterogeneity of cancer genomes [2,8,9] makes this kind of method sometimes ineffective because patients even from the same tumor type can have different driver mutations.

Further studies revealed that the acquisition of tumorigenic properties, such as cell proliferation, angiogenesis, or metastasis are mainly due to disruption of some cellular signaling and regulatory pathways [10,11]. Driver mutations either directly target such biological pathways or tend to cluster within closely knitted network modules which are closely linked to specific biological pathways [12,13]. Thus, identification of mutated driver pathways or core modules is of primary importance for understanding cancer initiation and progression. Moreover, a great deal of investigation indicates that genes in the driver pathway or core module usually cover a large number of samples and exhibit mutual exclusivity, these two criteria are commonly used in the pathway or module based methods. For example, Ding *et al. *[8] and Jones *et al. *[9] analyzed known pathways for enrichment of somatic mutations, Boca *et al. *[14] and Efroni *et al. *[15] detected known pathways which are significantly mutated across the patients, and Cerami *et al. *[16] and Ciriello *et al. *[17] identified oncogenic network modules by using somatic mutation and the human reference network. Although the priori knowledge (such as protein-protein interactions (PPI) and signal transduction pathways) can provide some useful information for the detection of driver mutations, the incompleteness of the human PPI network and the existence of many unknown pathways may limit the wide application of such methods in some extent. Recently, methods and algorithms were developed for *de novo *discovery of mutated driver pathways and functional modules in tumors based solely on cancer genomic data [18-20].

On the other hand, somatic mutations and CNVs in cancer genomes frequently perturb the expression level of affected genes and thus disrupt pathways controlling normal growth. Genes in the same pathway usually have similar gene expression profiles and thus can coordinately achieve a particular function [21]. Several studies have demonstrated the necessity of integrating gene expression information to identify candidate driver genes and driver pathways [19,22,23].

In this study we present an integrative method, called iMCMC (**i**dentify **M**utated **C**ore **M**odules in **C**ancer) that integrates gene sequence and expression information to identify mutated core modules in cancer. A typical character of iMCMC is that it uses only cancer genomic data without any prior knowledge such as PPI networks and known pathways. First, somatic mutations and CNVs are used to generate a mutation network, similarly an expression network is obtained from the gene expression profiles. Then, an integrative molecular network is constructed by combining these two networks (i.e., integrating the three different kinds of data). Finally, an optimization model is used to identify coherent subnetworks (modules), which are further assessed by statistical tests. These are key contributions of our approach. The main consideration is that cooperative dysregulation of gene sequence and expression may contribute to cancer formation and progression. Furthermore, cellular networks contain functional modules, and tumors usually target specific modules critical to their growth. More importantly, our weighted integrative network is constructed to take into consideration possible features of genes in the driver pathways or core modules: large coverage, mutual exclusivity, strong influence of a gene's mutation on other genes, and high correlation of gene mutation-correlated expressions. All these factors are reflected in the vertex weight or edge weight of the integrative network. Applying iMCMC to the TCGA glioblastoma multiforme (GBM) and ovarian carcinoma data, we identified five and two mutated core modules, respectively. In the GBM data, the involved pathways include parts of the RB signaling and RTK signaling pathways (*CDKN2B, CDK4*; *EGFR, NF1 *), and in the ovarian carcinoma data a recurrent mutated module related to cell cycle and DNA repair (*CCNE1, MYC, RAD52 *) is revealed. Importantly, most of the implicated genes are oncogenes or tumor suppressors previously reported to be related to cancer pathogenesis (others include *TP53, PTEN, RB1, MDM2 *for GBM, and *KRAS *for ovarian carcinoma). Furthermore, to investigate the possible role of gene expressions or CNVs for the identification of mutated core modules, we also performed iMCMC on the datasets consisting of somatic mutations combined with CNVs or gene expressions. The results indicate that each indeed provides extra useful information to the original data for module detection. To conclude, as a generally applicable methodology, iMCMC can identify not only some known pathways but also provide candidate pathways or core modules recurrently perturbed in cancer for further studies.

## Results and discussion

### Overview of our method

**Figure 1:**
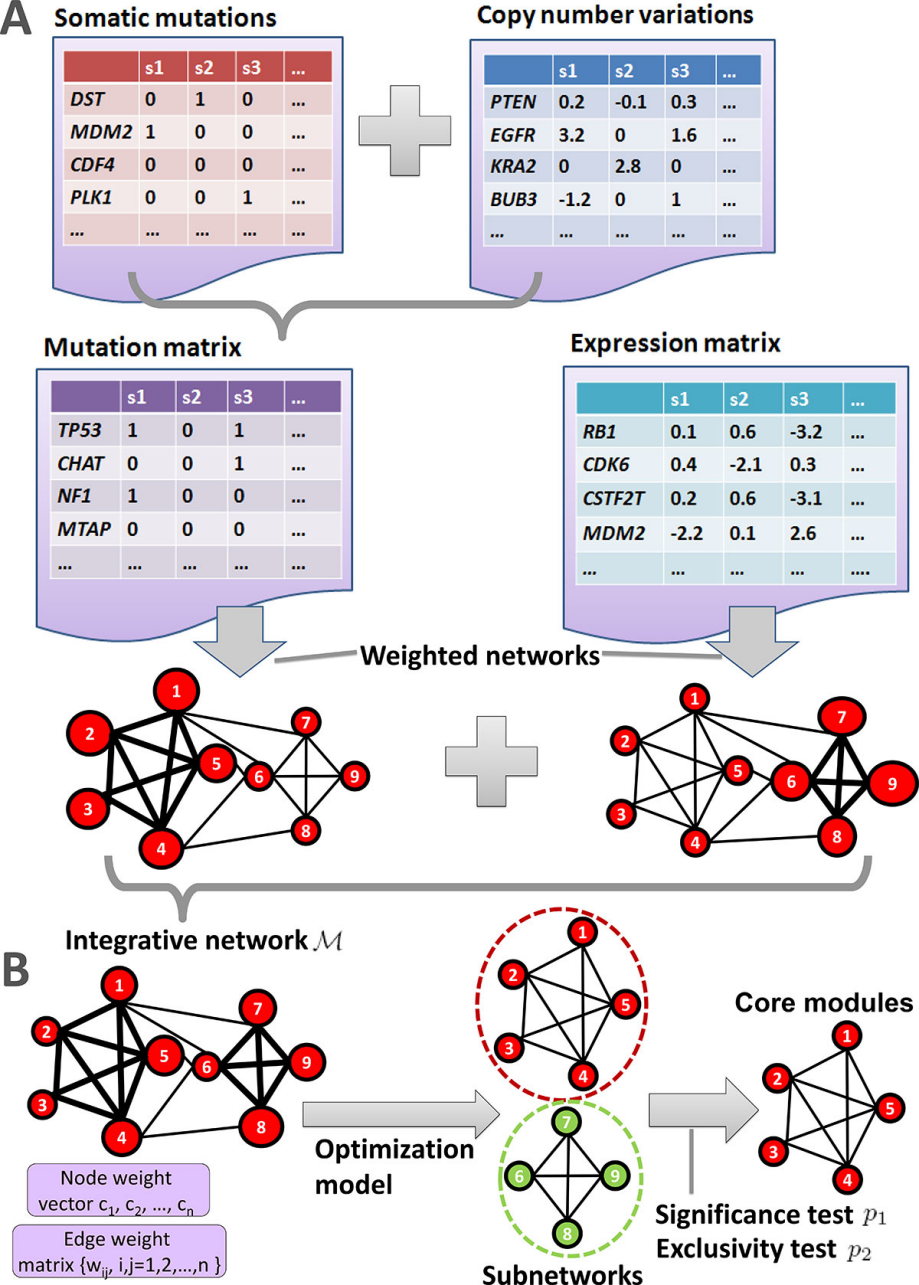
**Schematic overview of the integrative iMCMC method**. (A) Illustration of the construction of two networks with weighted vertices and weighted edges. Firstly, a mutation matrix can be obtained by combining the somatic mutations and CNVs, and an expression matrix is obtained from the gene expression profiles. Then, the weighted mutation network and expression network are constructed from these two matrices. (B) These two networks are combined to get an integrative network M , for which an optimization model is used to identify the most coherent subnetworks. Finally, we obtained the core modules for tumors by filtering with significance and exclusivity tests, for which two *p*-values *p*_1 _and *p*_2 _are calculated, respectively.

Three kinds of data including somatic mutations, CNVs, and gene expressions were used in this study. All data were downloaded from the TCGA website (https://tcga-data.nci.nih.gov/tcga/). The proposed iMCMC method for identification of mutated core modules contains six steps. A schematic overview of iMCMC is displayed in Figure 1. For additional details please refer to the **Materials and methods **section.

**Step 1**: A mutation matrix is obtained by combining somatic mutations and CNVs, and an expression matrix is generated from gene expression profiles.

**Step 2**: A mutation network and an expression network are constructed based on the mutation and expression matrices, respectively.

**Step 3**: These two networks are integrated into an integrative network.

**Step 4**: Coherent subnetworks (modules) are identified using an optimization model.

**Step 5**: A random test is performed to assess significance of the selected subnetworks, for which a *p*-value *p*_1 _is obtained.

**Step 6**: Finally, a Markov chain Monte Carlo permutation strategy is adopted to test mutual exclusivity of the subnetworks, and a *p*-value *p*_2 _is calculated.

In the end, core mutated modules can be obtained if the selected subnetworks pass the last two statistical assessments.

### Application to glioblastoma multiforme (GBM)

Among the glioblastoma dataset obtained from TCGA, DNA copy number variations are present in 169 samples, gene expression profiles in 202 and nucleotide sequence aberrations in 135 samples. Using the construction procedure of the integrative network 93 genes were left in the integrative network M  (notice that some of these are metagenes - genes that are mutated in the same samples). These genes are present in 90 samples common to all three kinds of data. Five core modules are obtained by performing iMCMC on M , where λ = 1 is used (see **Materials and methods**).

The first module consists of *CDKN2A *and *CYP27B1 *and covers 60 GBM samples. Initially, five genes including *CDK4, CDKN2A, CDKN2B, CYP27B1*, and *MTAP *were detected. This group of genes has a significant *p*-value of *p*_1 _< 0.001 and the exclusivity *p*-value of *p*_2 _= 1. After sequentially removing some co-occurring genes, we obtained *CDKN2A *and *CYP27B1 *with *p*_2 _*<*0.001. Previously, *CDKN2B *and *CYP27B1 *were identified as the most frequently sampled pair for GBM [18]. Both *CDKN2A *and *CDKN2B *are tumor suppressors located on 9p21.3-22.3 which is a common homozygous deletion region on the human chromosome. These two genes mutate almost simultaneously in all samples, so they have a very low exclusivity value (0.06), and it is not contradictory for us to identify *CDKN2A *instead of *CDKN2B *in the module for further analysis. *CDKN2A *encodes protein *p16*, which is a tumor suppressor protein with an important role in cell cycle regulation [24]. Mutations in *CDKN2A *are associated with increased risk in a wide range of cancers. Especially, recent studies showed that *CDKN2A *in high-grade glioma tissues was significantly down-regulated than in low-grade glioma tissues [25], which indicates that *CDKN2A *may be involved in malignant glioma carcinogenesis. *CYP27B1 *plays an important role in normal bone growth, calcium metabolism, and tissue differentiation. Gene amplification and mRNA splice variants of *CYP27B1 *in human glioblastoma were also previously reported [26].

**Figure 2:**
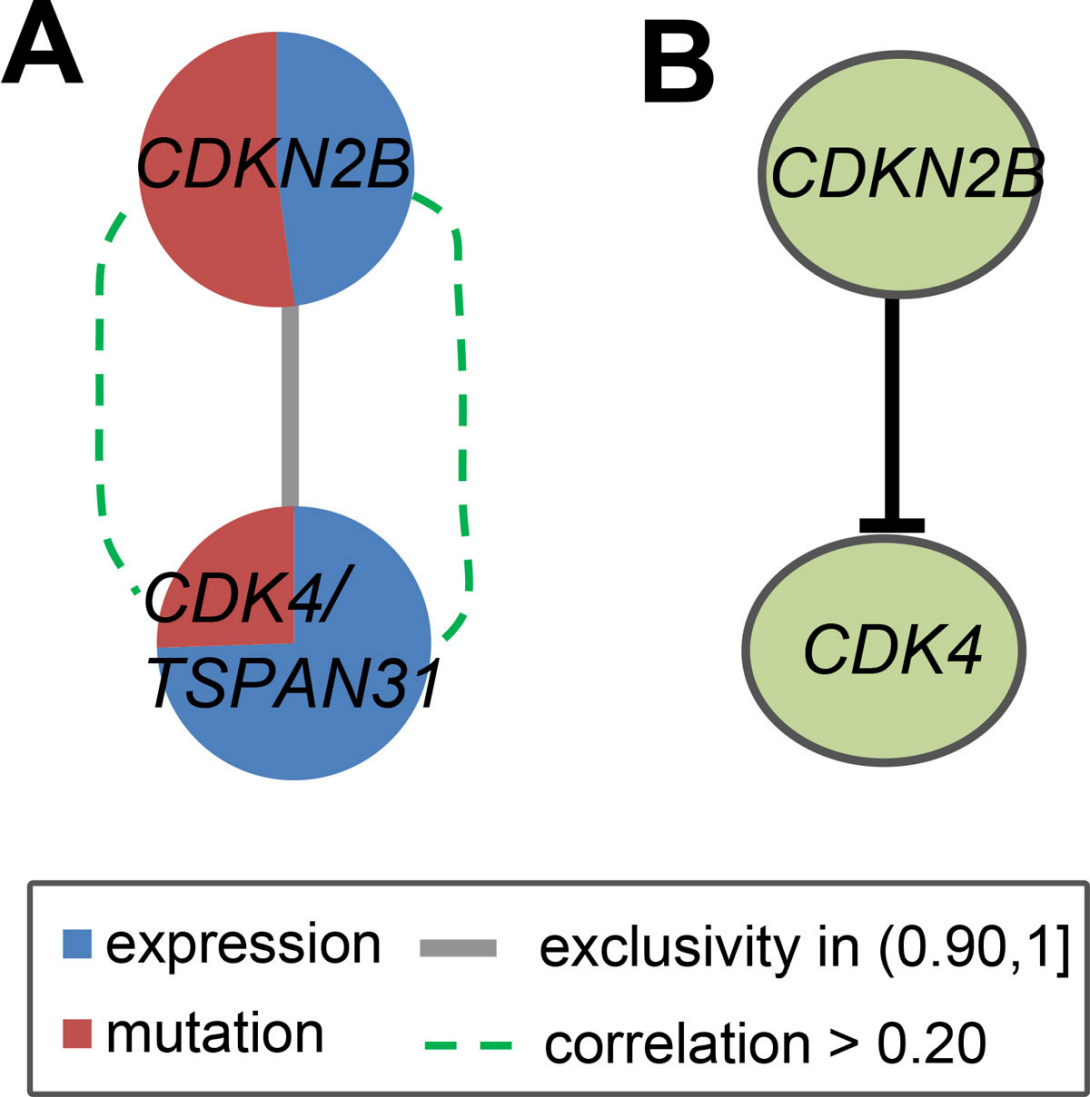
**The second module identified by iMCMC and the corresponding known pathway in the GBM data**. (A) Red and blue colors in the vertex represent the proportion of gene's coverage and the influence of its mutation status on other genes. Thick gray line shows that gene *CDKN2B *and the metagene *CDK4/TSPAN31 *are approximately exclusive (with exclusivity more than 0.90), whereas the dashed green curve represents existence of relative correlation between the expressions of the corresponding pair of genes. It is similar for the following figures. (B) *CDK4 *is suppressed by *CDKN2B *in the RB signaling pathway, where the interaction is as reported in [2].

The second module is obtained by removing *CDKN2A *and *CYP27B1 *from the integrative network M  and performing iMCMC on the remaining genes (Figure 2). *CDKN2B *and a metagene including *CDK4 *and *TSPAN31 *were identified with a coverage rate of 63/90. This module is significant and the genes *CDKN2B *and *CDK4/TSPAN31 *are mutually exclusive with *p*_1 _*<*0.001 and *p*_2 _*<*0.001. Several studies have found that variants of *CDKN2B *are associated with high-grade glioma susceptibility [27]. Feng *et al. *[28] made an integrated analysis of multiple kinds of data at 9p21.3 in glioblastoma and showed that the complete loss of 9p21.3 and low *CDKN2B *expression were associated with worse prognosis for both tumor progression/recurrence-free survival. The functional importance of *CDK4 *in astrocytic tumourigenesis, particularly during the later stages of tumor progression has been reported [29]. This gene has also been a putative prognostic marker and related to the survival of GBM patients [30,31]. As an oncogene, *CDK4 *is suppressed by *CDKN2B *in the RB signaling pathway (Figure 2B). The gene *TSPAN31 *is thought to be involved in growth-related cellular processes, because the encoded protein mediates signal transduction events thus plays a role in the regulation of cell development, activation and growth. *TSPAN31 *is associated with tumorigenesis although there is no report about its relationship with GBM. However, *TSPAN31 *was also found highly amplified in a number of GBM patients elsewhere. Here *TSPAN31 *and *CDK4*, as a metagene, mutate in the same samples, and both are relatively correlated to *CDKN2B *(Figure 2A).

A more detailed explanation of Figure 2A will help demonstrate the advantage of our framework, which can further enable understanding of how the three kinds of data are integrated and utilized for identification of the module. Both mutation (including somatic mutations and CNVs) and expression information exists not only in the edge but also in the vertex. In the vertex of Figure 2A, these correspond to the red and blue parts, respectively, which represent the proportion of coverage and the influence of mutation status on other genes while along the edge they point to mutual exclusivity and gene expression correlation respectively. This and other figures show that exclusivity in the detected module is always large due to the application of mutual exclusivity test in iMCMC. Although the expression correlation is not very high, the influence of some genes on other genes calculated from their expression is sometimes heavily utilized in the vertex. Therefore, we presume that gene expression indeed plays an important role in the identification of mutated core modules which is reflected either in the edge or in the vertex of the integrative network.

Performing iMCMC after removal of the foregoing two modules from M  results in the third module with three genes (*TP53, PTEN *and *MTAP *) and the fourth module including three other genes (*EGFR, NF1 *and *MDM2 *) and a metagene (*CHAT/SLC18A3 *). Both modules are highly significant (*p*_1 _*<*0.001 and *p*_2 _*<*0.001) and cover 70 and 46 GBM samples, respectively. Finally, our method identifies a module including *RB1 *and a metagene *DKK1/PRKG1/CSTF2T *significant at *p*_1 _*<*0.001 and *p*_2 _= 0.02 levels. Besides these no other significant modules are detectable.

In the third module, both *TP53 *and *PTEN *are important tumor suppressors [32]. When *PTEN *is mutated or deleted its enzymatic activity will be inactivated which may lead to increased cell proliferation and reduced cell death. Several studies indicate that concomitant inactivation of *TP53 *and *PTEN *promoted the development of glioblastoma. This co-operative nature was also validated in adult brain of mature mice [33]. The gene *MTAP *encodes an enzyme that plays a major role in polyamine metabolism and is important for the adenine and methionine salvage pathway [34]. A number of studies indicate that *MTAP *deficiency is a common occurrence in various cancers including glioblastomas, non-small cell lung cancer, melanoma, pancreatic and endometrial cancer [35,36]. Here *MTAP *not only has stronger exclusivity but also higher gene expression correlation with *TP53 *than *PTEN *in the identified module.

**Figure 3:**
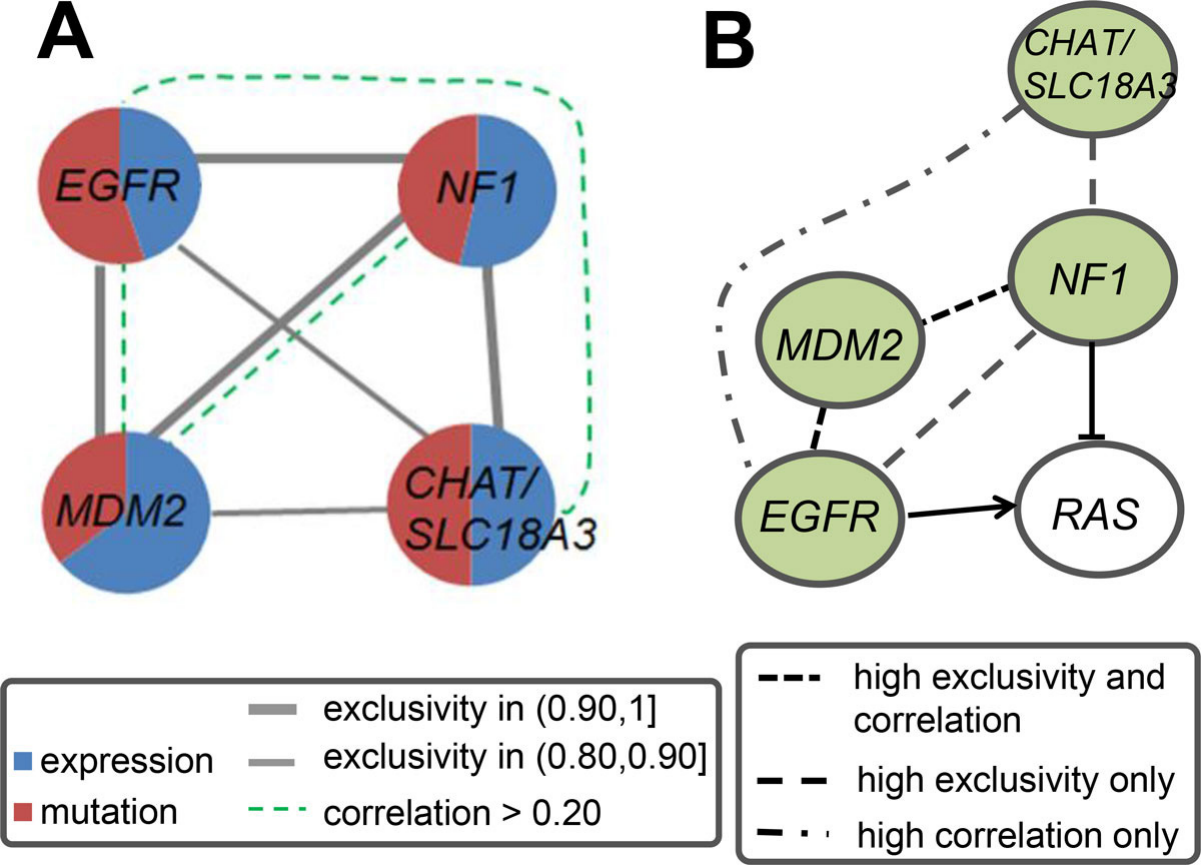
**The fourth module identified by iMCMC in the GBM data and locations of the detected genes in known pathways**. (A) Mutual exclusivity between any pair of genes is greater than 0.80 in this module. Thick gray line denotes a value more than 0.90 and thin line represents a value between 0.80 and 0.90. (B) The detected genes *EGFR *and *NF1 *are in the RTK signaling pathway, where the interactions are as reported in [2]. The dense dashed line represents pair of genes with exclusivity more than 0.90 and correlation larger than 0.20. The loosely dashed line represents exclusivity more than 0.90 only, and the dot-and-dash curve represents correlation larger than 0.20 only.

The genes *EGFR *and *NF1 *in the fourth module are involved in the RTK signaling pathway (Figure 3B), which is one of the core pathways altered in the development of GBM [2]. *NF1 *is a human glioblastoma suppressor gene while *EGFR *is frequently activated in primary glioblastomas. Both have been used as biomarkers for the identification of the glioblastoma subtypes [37]. Amplification of *MDM2 *or increased expression occurs in many tumors [38]. Although *TP53 *and *MDM2 *often form a negative feedback loop by *MDM2 *inhibiting *TP53 *activity which results in transcriptional up-regulation of *MDM2 *expression, functions of *MDM2 *independent of *TP53 *have also been identified. For example, Biernat *et al. *demonstrated the molecular mechanism of *MDM2*'s escape from *TP53*-regulated growth control [39]. The gene *CHAT *encodes an enzyme which catalyzes the biosynthesis of the neurotransmitter acetylcholine. *SLC18A3 *is located within the first intron of *CHAT *and aids in the transport of acetylcholine, synthesized by *CHAT*, into secretory vesicles for release into the extracellular space. *CHAT *is presently the most specific indicator available to monitor the functional state of cholinergic neurons in the central and peripheral nervous systems [40]. Central cholinergic neurons are involved in several neurodegenerative diseases such as Alzheimer's disease and amyotrophic lateral sclerosis. Abnormalities of *CHAT *in the brain have also been demonstrated in schizophrenia and sudden infant death syndrome. In the fourth module, *MDM2 *and *EGFR *as well as *MDM2 *and *NF1 *are highly exclusive with high correlation in expression (Figure 3). Moreover, high exclusivity or correlation is also observed between *NF1 *and *EGFR*, *NF1 *and *CHAT/SLC18A3 *as well as *EGFR *and *SLC18A3*, respectively.

### Application to ovarian cancer

The ovarian carcinoma dataset from TCGA describes DNA copy number variations in 559 high-grade serous ovarian adenocarcinomas, gene expression profiles in 489 tumors and DNA sequence aberrations in coding genes of 320 tumors. After preprocessing, we obtained 371 genes in the integrative network M  in 311 samples. Here, some genes merged as metagenes.

We notice that *TP53 *is the most commonly mutated gene and is present in more than 80% of the high-grade serous ovarian carcinomas while all other genes are mutated in less than 27% of samples. In addition, analysis of *TTN *mutations indicates that these are likely to be artifacts [41]. Considering the prevalence of *TP53 *mutation and the possible inaccuracy of *TTN *mutations, we removed these two genes from M  and performed iMCMC on the remaining genes for which two mutated core modules are identified.

**Figure 4:**
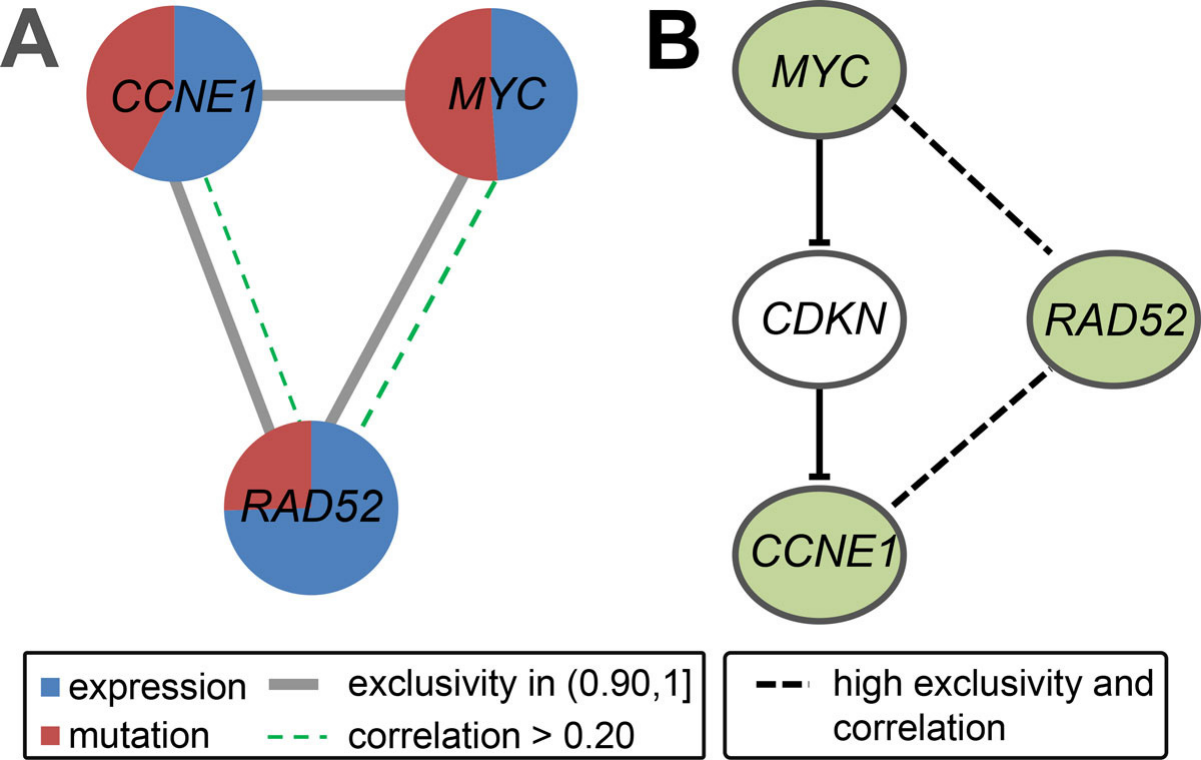
**The first module identified by iMCMC in the ovarian carcinoma data and locations of the detected genes in known pathways**. (A) All pairs of genes in this module are highly mutually exclusive with values more than 0.90. (B) The detected genes *MYC *and *CCNE1 *are in the cell cycle pathway. The dense dashed line represents pair of genes with exclusivity more than 0.90 and correlation larger than 0.20. *RAD52 *plays a central role in genetic recombination and DNA repair, and is not only highly exclusive but also correlated with *MYC *and *CCNE1*.

The first module consisting of three genes, *CCNE1, MYC *and *RAD52*, is statistically significant with *p*_1 _*<*0.001 and *p*_2 _*<*0.001. This module is approximately exclusively mutated in 150 samples. *CCNE1 *and *MYC *are two important genes engaged in cell cycle progression (Figure 4B). The gene *CCNE1 *is essential for the control of the cell cycle at the G1/S transition. In many tumors overexpression of this gene results in chromosome instability that may contribute to tumorigenesis [42]. Nakayama *et al. *demonstrated that amplification of *CCNE1 *is related to poor survival suggesting that *CCNE1 *can be a potential therapeutic target in the treatment of ovarian cancer [43]. *MYC *is a strong proto-oncogene that codes a transcription factor and is often found to be constitutively (persistently) expressed in many types of cancers [42]. This leads to the unregulated expression of many genes (presumably through DNA over-replication), some of which are involved in cell proliferation and result in cancer formation [44]. The gene *RAD52 *is involved in double-stranded break repair and plays a central role in genetic recombination and DNA repair. Experiments by Schilddraut *et al. *provide evidence for an association between several genes in the DNA repair and response pathways and risk of invasive serous ovarian cancer [45]. In addition to genes with strong support associations, the study is also supportive of associations between three SNPs in *RAD52 *and invasive serous ovarian cancer. More importantly, *RAD52 *in the current module is not only highly exclusive but also correlates with *MYC *and *CCNE1*.

The second module consists of *KRAS *and *PPP2R2A *with *p*_1 _*<*0.001 and *p*_2 _= 0.05, covering 77 samples. As an Oncogene, *KRAS *is an important signal transducer involved in the regulation of various cellular responses during cell proliferation, differentiation, and survival. Mutations in *KRAS *frequently occur in cancer cells such as specific ovarian cancer subtypes [46] and indicate poor prognosis and increased resistance to some cancer therapies [47]. The protein encoded by *PPP2R2A *is also implicated in the negative regulation of cell growth and division, and is associated with a variety of regulatory subunits. Although *PPP2R2A *has not been directly implicated in tumorigenesis, several findings suggest that deregulation of *CHEK2 *and/or *PPP2R2A *has pathogenic effects in at least a subset of germ cell tumors in childhood teratoma [48].

### Analysis using only somatic mutations and CNVs

**Figure 5:**
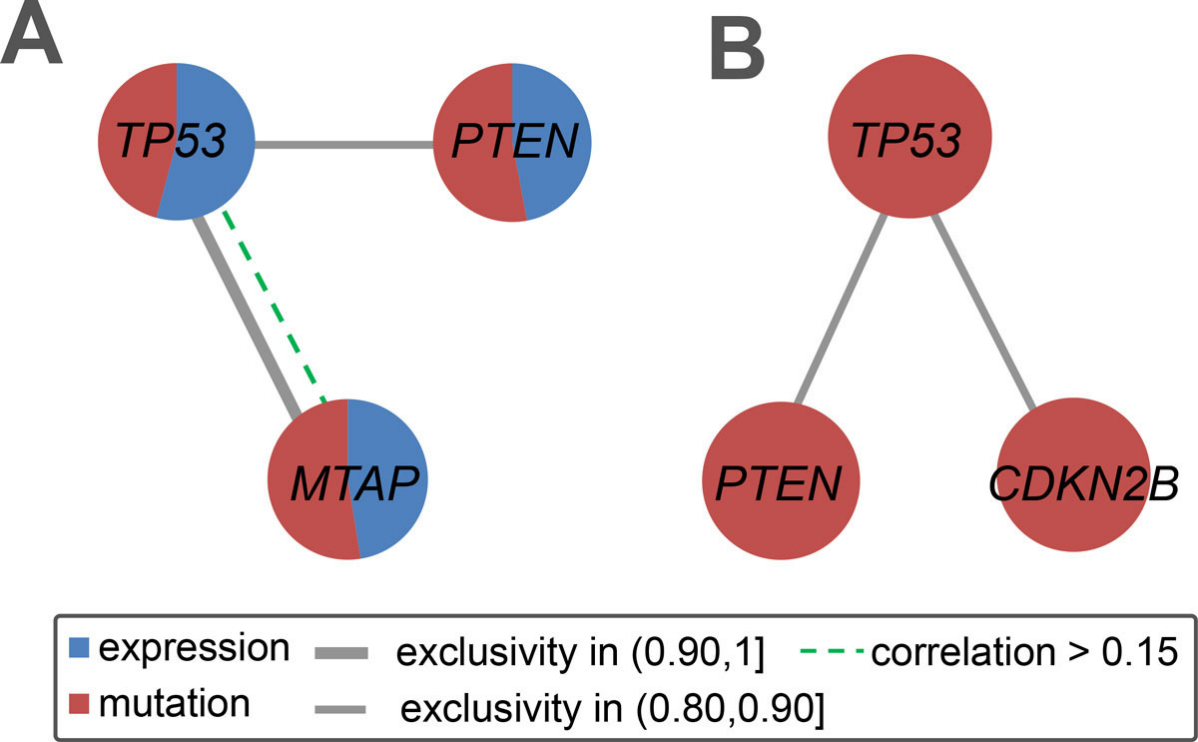
**A comparison of the GBM data with or without gene expressions**. (A) When all three kinds of data are used, the identified module has high exclusivity and relative correlation between *TP53 *and *MTAP*. (B) When gene expressions are not used the detected module is not as exclusive as before.

To further investigate if gene expression provides useful information for the identification of mutated core modules in cancer, we analyzed data only from somatic mutations and CNVs. In this case only one significant module is detected for each dataset. In GBM, the module contains three genes *CDKN2B, PTEN *and *TP53*. Compared to the module containing *PTEN, TP53 *and *MTAP *identified using all three kinds of data, the current module has lower exclusivity between several pairs of genes (Figure 5). In the ovarian carcinoma data the module consists of *CCNE1 *and *MYC*. Interestingly, *RAD52 *is not detected, although it has a high correlation and very high exclusivity both with *CCNE1 *and *MYC *(Figure 4). All these indicate that gene expression is helpful for the identification of biologically mutated core modules in cancer.

### Integration of somatic mutations and gene expressions data

Recently, core modules were detected in the GBM data without using CNV information [49]. In this case three modules were identified which are significantly mutually exclusive (Table 1). A slightly different strategy was adopted in [49] for data integration: more weight is given to somatic mutations than gene expressions (i.e., *k *= 2 was used in the integrated model); a smaller threshold is selected to detect a bigger subnetwork in the optimization algorithm; and the statistical test for mutual exclusivity is also slightly different.

**Table 1:** Modules identified when only somatic mutations and gene expressions are used

**Module**	**Gene**	***p*-value for exclusivity**	**Related to GBM**	**Reference**
1	*EGFR, NF1*, *PTEN, PIK3R1, TP53*	0.01	all the genes	[49] and the references wherein
2	*COL6A2, DST*, *ERBB2, PIK3CA, RB1*	*<*0.001	all except *DST*	same as above
3	*PRAME, SYNE1*	*<*0.001	both genes	same as above

Several oncogenes or tumor suppressors such as *PTEN, TP53, EGFR *and *NF1 *were also detected when these two kinds of data are used for GBM. These four genes are involved in the RTK/RAS/PI(3)K signaling pathway, which is one of the core pathways altered in the development of glioblastoma and was deduced by the TCGA Research Network [2]. It should be noted that because of the lack of CNV data, several genes including *CDKN2A, CDKN2B, CDK4 *and *MDM2 *were not identified.

## Conclusions

In this paper, iMCMC is presented to integrate somatic mutations, CNVs and gene expressions to detect mutated core modules in cancer. Unlike previous approaches exploring pathways or modules, iMCMC does not use any prior information such as human PPI networks and known pathways. We apply iMCMC to the GBM and ovarian carcinoma datasets and identified five and two mutated modules respectively. Many of the detected genes have been reported to be implicated in carcinogenesis and some modules are involved in known pathways. For example, *CDKN2B *and *CDK4 *as well as *EGFR *and *NF1 *are involved in the RB and RTK signaling pathways, respectively and the *CCNE1, MYC *and *RAD52 *module in ovarian carcinoma is involved in cell cycle.

For further improvement of the integrative network M  and the optimization algorithm in iMCMC, two parameters, i.e., *k *and *λ*, should be further explored. A typical feature of our method is to employ two parameters to balance not only different sources of data but also the vertices and edges of the weighted network. This provides flexibility for using the method because one can choose different parameters to emphasize on specific factors. It should be noted that different choices for parameters may result in slightly different results. This needs further consideration in practice based on actual data.

For comparison, we tested the proposed method on datasets integrating only two kinds of data, i.e., somatic mutations and CNVs or expressions. The results indicate that gene expressions or CNVs indeed provide extra useful information to the original data for the identification of mutated core modules in cancer.

In conclusion, our findings provide several candidate core modules recurrently perturbed in GBM or ovarian carcinoma for further studies. Our integrative method, iMCMC, will be a helpful complementary tool in the identification of cancer pathways and as a general methodology with practical significance, it has a potential to be employed in cancer research.

## Materials and methods

### Data sets

The GBM and ovarian carcinoma data were downloaded from TCGA website (https://tcga-data.nci.nih.gov/tcga/) in December, 2011. We used three kinds of data: somatic mutations, DNA copy number variations (CNVs), and gene expressions. We considered only the data from level 3. The GBM dataset contains CNVs in 1269 genes spanning 169 glioblastoma samples, gene expression profiles in 11861 genes in 202 samples and nucleotide sequence aberrations in 343 genes in 135 samples. For the ovarian carcinoma dataset, these three kinds of data are in 966 genes in 559 samples, 11864 genes in 489 samples and 8431 genes in 320 samples, respectively. All these data are primary materials required to construct the integrative network for further analysis. First, a matrix *A*_0 _is generated by identifying common samples with somatic mutations and CNVs and merging their genes over the common samples. *A*_0 _is binary: if any mutation occurs in a given gene in a particular sample or if the given gene is in a statistically significant variation region of the particular sample, which is determined by GISTIC [50], then the mutation is assigned the number 1; if these criteria are not met then 0 is assigned. A mutation matrix *A *is then obtained by reducing the size of *A*_0 _by combining genes that are mutated in the same samples into larger 'metagenes'. An expression matrix *B *is obtained by using the method described previously [37]. *B *is a real matrix with each of its entries representing relative expression of a given gene in a particular sample. For all these matrices, rows and columns correspond to samples and genes, respectively.

The main idea of this study is to integrate three kinds of data resources described above via a network framework and identify mutated core modules in cancer by an optimization model and the following statistical tests. The preliminary version of this method was recently proposed in [49] for GBM somatic mutation and gene expression integrative analysis. For the completeness of this paper we describe the approach with some improvements in the following.

### Construction of an integrative network M 

With the above data, we constructed an integrative network based on which an optimization model can be built to detect oncogenic modules and pathways. The construction procedure contains three steps.

#### The network based on gene expression

In this step a network based on gene expression called *Expression Network *(denoted by **EN**) is constructed. **EN **is weighted both for its edges and vertices, where each vertex denotes a gene, and each edge is the correlation between expressions of two vertices (genes). Weight of each vertex reflects the extent of the influence of a gene mutation on the expression of other genes.

We notice that genes in *A *and *B *may be different and so the common genes are identified first. Let (*G*_1_, *S*_1_) and (*G*_2_, *S*_2_) be the sets of genes and samples contained in the two matrices, respectively. *G*_0 _and *S *are set as *G*_0 _= *G*_1 _∩ *G*_2 _and *S *= *S*_1 _∩ *S*_2_. For any gene *i *∈ *G*_0_, the samples in *S *are classified into two groups according to the binary mutation vector of *i *from the mutation matrix *A*, and the corresponding numbers of samples are denoted as $n_{i}^{\left(1\right)}$ and $n_{i}^{\left(2\right)}$, respectively. Moreover, based on the elements in *A *and *B *we set $e_{i}^{\left(1\right)}=\left\{b_{ki}:a_{ki}=1,k\in S\right\}$ and $e_{i}^{\left(2\right)}=\left\{b_{ki}:a_{ki}=0,k\in S\right\}$, and so a mutation-correlated expression vector $e_{i}=\left(e_{i}^{\left(1\right)},e_{i}^{\left(2\right)}\right)$ can be obtained. Then *p*-values for all genes in *G*_2 _are calculated using the program *mattest *in MATLAB toolbox to evaluate the extents of differential expression of these genes related to *i*'s mutation status. A prerequisite for this procedure is a minimum number of 2 for samples in the two groups. Therefore, the vertex set of the expression network **EN **is *G *where

$$G=\left\{i\in G_{0}:n_{i}^{\left(1\right)}\geq 2,n_{i}^{\left(2\right)}\geq 2\right\}.$$

For any gene *i *∈ *G*, the vertex weight of **EN **can be defined as:

$$f_{i}=1-1/d\overset{d}{\underset{r=1}{\sum }}p_{r},$$

where *d *is the number of genes in *G*_2_, and *p_r _*is the *p*-value of differential expression of gene *r *relative to *i*'s mutation status. This means that smaller the *p*-values stronger the influence of a gene mutation on others. That is, it is more likely to be a driver that should be given greater weights.

For any two genes *i *and *j *in *G*, the edge weight *u_ij _*is defined as the absolute value of Pearson correlation between *e_i _*and *e_j _*among the samples in *S*. Note that corresponding to metagene, weights of the vertex and edge in the expression network are obtained from averages of the values of related genes.

#### The network based on somatic mutations and CNVs

Based on the mutation matrix *A *generated from somatic mutations and CNVs, a *Mutation Network *(**MN**) can be constructed. To hold the same vertex set as in the expression network **EN**, the same gene set *G *is used to construct **MN**. For any gene *i *∈ *G*, *m_i _*denotes the number of mutations in *i *across the samples in the mutation matrix *A*, i.e., $m_{i}=\sum_{r}a_{ri}$. The vertex weight is defined as

$$h_{i}=m_{i}/m,$$

where *m *is the number of all samples in *A*. For any pair of genes *i *and *j *in *G*, the edge weight *v_ij _*is defined as the number of samples in which exactly one of the pair is mutated divided by the number of samples in which at least one of the pair is mutated in *A*. The vertex weight is a measure of mutation coverage and the edge weight is a measure of mutual exclusivity.

#### The integrative network

An integrative network M  can be obtained by synthesizing the expression network **EN **and the mutation network **MN**.

We observed that in **EN **or **MN **the vertex and edge weights have different measurement levels. To balance these two terms, we defined *f *= max *f_i _*and *u *= max *u_ij _*in **EN **and similarly, *h *= max *h_i _*and *v *= max *v_ij _*in **MN**. We set *ξ *= *u/f*, and *η *= *v/h*. Let *F *= {*f_i_*} and *U *= {*u_ij_*} denote the sets of vertex weights and edge weights in **EN**, respectively (similarly, *H *= {*h_i_*} and *V *= {*v_ij_*} in **MN**). Then *U *and *ξF *(similarly, *V *and *ηH*) have balanced values.

While integrating the two networks more importance can be given to **MN **than **EN **when gene expression values are considered to contain noises. Thus a parameter *k *is introduced to reflect the relative importance of **MN **relative to **EN**. Set *δ *· (*u/v*) = *k*, then *δ *= *k/*(*u/v*). In this paper *k *= 1 is used.

The integrative network M  with edge weights *w_ij _*and vertex weights *c_i _*can be defined as follows:

(1) $$w_{ij}=\delta .\;u_{ij}+v_{ij},\;c_{i}=\delta \xi \cdot f_{i}+\eta \cdot h_{i},$$

i,j=1,⋯,n,

where *n *is the number of genes in *G*. From the above discussion it is clear that *ξ *and *η *can be directly determined by the **EN **and **MN **networks, which is also similar for *δ *once *k *is preassigned.

### An optimization model for detecting coherent subnetworks

For the integrative network M , our goal is to extract some modules (subnetworks) with high weights in both edges and vertices. We used the previously reported optimization model [51] for this purpose. With *w_ij _*and *c_i _*defined as in Eq. (1), the model is as follows:

(2) $$\begin{array}{c} \text{max}\;\sum_{i}\sum_{j}w_{ij}x_{i}x_{j}+\lambda c_{i}x_{i},\\ s.t.\;\;x_{1}^{\beta }+x_{2}^{\beta }+\cdots +x_{n}^{\beta }=1,\\ \;\;\;\;x_{i}\geq 0,i=1,\cdots \;,n, \end{array}$$

where the *n*-dimensional non-negative vector *x *= (*x*_1_, *x*_2_, ..., *x_n_*), determined by solving the optimization model, represents the degree of each vertex that belongs to a specific subnetwork. The first term in the objective function measures the interconnectivity within the subnetwork, while the second term measures the degree of association between vertices and the subnetwork. In the model, a positive parameter *λ *is introduced to balance these two terms.

On the other hand, a trivial solution will be obtained when model (2) is unconstrained where all vertices from the original network can be included into the subnetwork, so a regularization constraint should be introduced to limit the number of vertices selected. This is the role of *β *which can adjust the strength of regularization applied to the variable *x *= (*x*_1_, *x*_2_, ..., *x_n_*). *β *= 2 is an attractive option in many cases since the optimization of a quadratic function over a sphere is polynomially solvable in contrast to general non-convex programming [52] but tends to select all vertices in the network to the final subnetwork. The *L*1-type constraint when *β *= 1, leads to a sparse solution, i.e., many of the entries in the final optimal solution *x *will be zeros [53]. In general, we use *β *= 1 in model (2) to extract small-sized subnetworks from a larger network.

The optimization model (2) can be easily solved by quickly finding a local maximum from a predetermined initial solution using the following iterative algorithm [51]:

(3) $$x_{i}^{t+1}={\left(x_{i}^{t}\frac{2{\left(WX\right)}_{i}+\lambda c_{i}}{2X^{T}WX+\lambda \sum_{i}c_{i}x_{i}^{t}}\right)}^{\frac{1}{\beta }},$$

where *W *= (*w_ij _*) is the *n × n *edge weight matrix, and $X={\left(x_{1}^{t},x_{2}^{t},\cdots \;,x_{n}^{t}\right)}^{T}$ is the *n*-dimensional solution vector at time *t*. Algorithm (3) is convergent and the non-zero entries in solution *x *(determined in practice as entries that are greater than the cutoff, 0.1 is used in this study) define a certain subnetwork (module). After one locally optimal solution is obtained, these corresponding vertices are eliminated from the network, and the whole procedure is then iterated, i.e., we solve another locally optimal solution and its corresponding subnetwork based on the new network.

### Significance test of the subnetwork (module)

We performed a random test to assess the significance of the results. For a selected subnetwork **SN **with *b *vertices, we obtained a quantity *C *which is the sum of all vertex weights and edge weights involved in **SN**. Then we randomly selected *b *vertices from the original network and obtained a similar quantity *CR*. This procedure is repeated 1,000 times and the number *r *of *CRs *which is larger than *C *can be calculated. The significant *p*-value of **SN **(denoted as *p*_1_) can be obtained from the quantity of *r *divided by 1,000.

### Mutual exclusivity test of the subnetwork (module)

After a subnetwork passes the significance test, the following step is performed to evaluate whether it exhibits a pattern of mutually exclusive genomic alterations. For this we used the 'switching permutation' method proposed by Ciriello *et al. *[17], which adopts a Markov chain Monte Carlo permutation strategy based on random network generation models.

Furthermore, although a subnetwork **SN **with *b *(*b >*2) vertices is not significantly mutually exclusive, we cannot exclude the possibility that one of its subsets is. In this case we can reduce the scale of the subnetwork sequentially, that is, a subset **SN***′ *of size *b − *1, contained in **SN**, is selected which is more likely to be significant among all the subsets of **SN **with *b − *1 vertices. This can be realized by choosing the paired vertices with the smallest exclusivity and removing one vertex with the smaller entry value *x *in the solution of (3). This process is repeated until either of the two conditions is reached: **SN***′ *is significantly mutually exclusive or *b *= 2. In this study, *p*_2 _denotes the exclusivity *p*-value for concise description.

## Competing interests

The authors declare that they have no competing interests.

## Authors' contributions

JZ and SZ conceived this project. JZ carried out the experiment and data analysis. JZ, SZ and YW carried out the biological analyses and wrote the manuscript. XSZ supervised this project. All authors read and approved the final manuscript.

## Declarations

The publication of this article is from the National Natural Science Foundation of China, No. 11001256, 11131009, and 61171007, the `Special Presidential Prize' Scientific Research Foundation of the CAS and the Foundation for Members of Youth Innovation Promotion Association, CAS.

This article has been published as part of *BMC Systems Biology *Volume 7 Supplement 2, 2013: Selected articles from The 6th International Conference of Computational Biology. The full contents of the supplement are available online at http://www.biomedcentral.com/bmcsystbiol/supplements/7/S2.
